# Chronic kidney disease in gout in a managed care setting

**DOI:** 10.1186/1471-2369-12-36

**Published:** 2011-08-03

**Authors:** Mahesh J Fuldeore, Aylin A Riedel, Victoria Zarotsky, Bhavik J Pandya, Omar Dabbous, Eswar Krishnan

**Affiliations:** 1 Abbott Laboratories, Health Economics & Outcomes Research, 100 Abbott Park Rd, North Chicago, IL, 60064, USA. Formerly at TAP Pharmaceutical Products Inc., now part of Takeda Pharmaceuticals International Inc; 2 OptumInsight, Health Economics & Outcomes Research, 12125 Technology Drive, Eden Prairie, MN, 55344, USA; 3 OptumInsight, Clinical Services, 22533 Jameson Drive, Calabasas, CA, 91302, USA; 4 Takeda Pharmaceuticals International, Inc., Global Health Economics Outcomes Research, One Takeda Parkway, Deerfield, IL 60015, USA; 5 Stanford University Medical School, Division of Immunology and Rheumatology, Stanford, CA, 94305, USA

## Abstract

**Background:**

To study the prevalence of chronic kidney disease (CKD) and its impact on allopurinol dosing and uric acid control among patients with gout.

**Methods:**

This was a retrospective study using data from a large US health plan. Claims and laboratory data were analyzed for enrollees from the health plan database from January 2002 through December 2005. Patients with gout were identified from pharmacy and medical claims data based on the presence of codes for gout medication or gout diagnosis. Severity of CKD was determined using the estimated glomerular filtration rate (eGFR). Allopurinol titration was defined as a change in average daily dose from first prescription to last prescription of ≥ 50 mg.

**Results:**

A total of 3,929 patients were identified for inclusion in this study, 39% of whom had CKD (based on having an eGFR < 90 mL/min/1.73 m^2^). Subjects with CKD were older (p < 0.01) and more likely to be women (p < 0.01), had a greater number of comorbid conditions (p < 0.01), and were more likely to be prescribed allopurinol (p < 0.01) compared to those with no CKD. The average starting dose of allopurinol was lower among those with CKD, and it decreased with worsening kidney function. Among the 3,122 gout patients who used allopurinol, only 25.6% without CKD and 22.2% with CKD achieved a serum uric acid concentration of < 6.0 mg/dL (p = 0.0409). Also, only 15% of allopurinol users had an upward dose titration (by ≥50 mg), but the average increase in dose did not differ significantly between those with and without CKD.

**Conclusions:**

About two out of every five patients with gout in this population had CKD. Allopurinol doses were not adjusted in the majority of CKD patients. Serum uric acid control in gout was poor among patients without CKD and even worse among those with CKD.

## Background

Gouty arthritis (gout) is relatively common in the general population, with an estimated prevalence of 4%, and it is associated with approximately 3.9 million ambulatory care visits per year [1,2]. Chronic kidney disease (CKD) is also common, affecting approximately 26 million adults in the United States over the age of 20 [3,4]. Specific lifestyles choices can influence the risk of gout, but lifestyle changes may not be sufficient to manage disease after the onset of gout [5].

Gout patients typically exhibit increased levels of serum uric acid. It is recommended that serum uric acid levels be lowered to a target of < 6 mg/dL to better manage gout symptoms, and to reduce synovial MSU crystals and acute gout attacks [6]. Uric acid is excreted primarily through the kidney, and any impairment of kidney function can result in hyperuricemia. Even among individuals with normal renal function, the presence of hyperuricemia has been correlated with future incidence of renal impairment and increased healthcare utilization and costs [7,8]. Treatment of gout in patients with CKD is complicated because full dosing of allopurinol, the uric acid-reducing medicine, requires adequate renal function to clear oxypurinol, its active metabolite [9]. It is recommended that patients with renal impairment should receive reduced doses of allopurinol, as they may be at increased risk for allopurinol-related toxicity [9]. However, suboptimal uric acid control has been associated with worsening renal function [10-12].

Studies suggest that the prevalence of both gout and CKD are likely to increase over time [3,4]. Although the vast majority of patients with gout in the United States seek treatment from primary care physicians [2], most studies of CKD in gout patients have been performed in other settings such as rheumatology clinics [12-15], dialysis databases [16], and renal transplant centers [17,18]. In the present study, a retrospective analysis was performed using a large US health plan database (and associated lab data) containing detailed information on gout diagnosis, urate-lowering therapy usage, and serum uric acid (sUA) levels. The objectives of this study were to determine the prevalence of CKD among a general population of gout patients in a normal practice setting, and to investigate the impact of CKD on allopurinol dosing and uric acid control.

## Methods

### Data source and study design

This was a retrospective analysis of medical and pharmacy claims data from a large managed health care plan in the United States affiliated with OptumInsight. Additional laboratory data for patients was also obtained for some analyses. During the period of this study (January 1, 2002 through December 31, 2005), the health plan covered approximately 14 million individuals, mainly in the United States. The data were used in a de-identified format that complied with the requirements of the Health Insurance Portability and Accountability Act (HIPAA).

### Patient identification

This study included commercial health plan members at least 18 years of age. Ideally, a gout diagnosis would be based on the demonstration of intracellular urate crystals in an appropriate clinical setting, or on clinical or survey criteria published by the American College of Rheumatology [19,20]. However, these approaches are not feasible in large retrospective studies such as this one. In this study, patients were considered to have gout and were selected for the study if they had a minimum of 2 qualifying claims (as described below) during the time period from January 2002 through December 2005. The first qualifying claim must have been a prescription claim for a gout medication (allopurinol, probenecid, colchicine, and/or sulfinpyrazone). The second qualifying claim may have been either a prescription claim for a gout medication or a medical claim with an International Classification of Diseases, Ninth Revision, Clinical Modification (ICD-9-CM) diagnosis code of 274.0. Patients were also required to have at least 1 serum creatinine laboratory result during the first 12 months of their follow-up period for study inclusion. Patients with evidence of dialysis, kidney transplant, and/or cancer during the study period were excluded from the analysis. To qualify patients were also required to be enrolled in the health plan for ≥ 365 days during a baseline period and for ≥ 365 days during a follow-up period.

### Measurements of kidney function and uric acid

The Modification of Diet in Renal Disease (MDRD) study equation was applied to patients' serum creatinine levels to estimate the glomerular filtration rate (eGFR) [21]. The definition and stages of CKD used for the study are based on staging described by the National Kidney Foundation Kidney Disease Outcomes Quality Initiative (NKF KDOQI™, as found at http://www.kidney.org/professionals/kdoqi/guidelines_ckd/toc.htm), with modifications as described below. All patients with eGFR ≥90 were defined as having no CKD (information on kidney damage in the presence of normal eGFR was unavailable, so NKF Stage 1 disease was rolled into the "no CKD category"). Patients with NKF Stage 2 disease (eGFR 60-89), NKF Stage 3 disease (eGFR 30-59), or NKF Stage 4 disease (eGFR 15-29) were defined as having CKD and assigned to the Stage 2 (mild disease) category, the Stage 3 (moderate disease) category, or the Stage 4 (severe disease) category, respectively. Patients with NKF Stage 5 disease (kidney failure, eGFR < 15) were rolled into the Stage 4 (severe disease) category, due to small sample size. As some patients may have had more than one eGFR value, determination of CKD stage was based on the last eGFR value in the follow-up period. Follow-up eGFR was used instead of baseline eGFR because not all patients had a baseline GFR, and as renal function deteriorates over time, the follow-up eGFR was expected to classify patients by their most severe disease indicator.

Patients' serum uric acid levels were obtained from laboratory data. In accordance with available guidelines, a serum uric acid concentration < 6 mg/dL (< 0.36 mmol/L) was designated as the desirable goal for gout patients receiving urate reduction therapy [22]. Patients were stratified by CKD stage, and the proportion of patients at goal was determined.

### Patient demographic and clinical characteristics

Patient demographic variables including age, gender, and geographic location were identified from the enrollment data. ICD-9-CM codes from medical claims were used to calculate scores for the Charlson-Deyo Comorbidity Index, a validated index of severity of comorbidity [23]. A validated comorbidity classification developed by Elixhauser and adopted by the US Agency for Healthcare Research and Quality (AHRQ) was used to examine gout-kidney disease relationships across comorbidity groups [24].

In a subset analysis, the proportion of gout patients receiving allopurinol prescriptions (medication fill rate) was evaluated. In addition, the allopurinol average daily dose was calculated at the time of initial observed dose and at the time of last observation. Patients were considered to have titrated their allopurinol dose if the absolute change in average daily dose from first prescription to last prescription was ≥ 50 mg. Additional analyses were conducted using a threshold of change in allopurinol dose of ≥100 mg to define therapy titration. Other medications for gout such as probenecid, colchicine, and sulfinpyrazone were used too infrequently in the study sample for meaningful analysis.

### Data analysis

For univariate and bivariate analyses, Student's *t*-test and Pearson's chi-squared test, respectively, were used. For multivariable analyses designed to determine factors associated with changes in allopurinol dose, a logistic regression with occurrence of a dose change of ≥50 mg/day was used as the dependent variable. Independent variables included severity of CKD, age, gender, region of plan membership, thiazide use, preexisting comorbid conditions, and length of allopurinol therapy. All analyses were conducted using the SAS (version 9.0) software program (SAS Institute, Inc., Cary, North Carolina).

## Results

### Patient selection and characteristics

**Figure 1:**
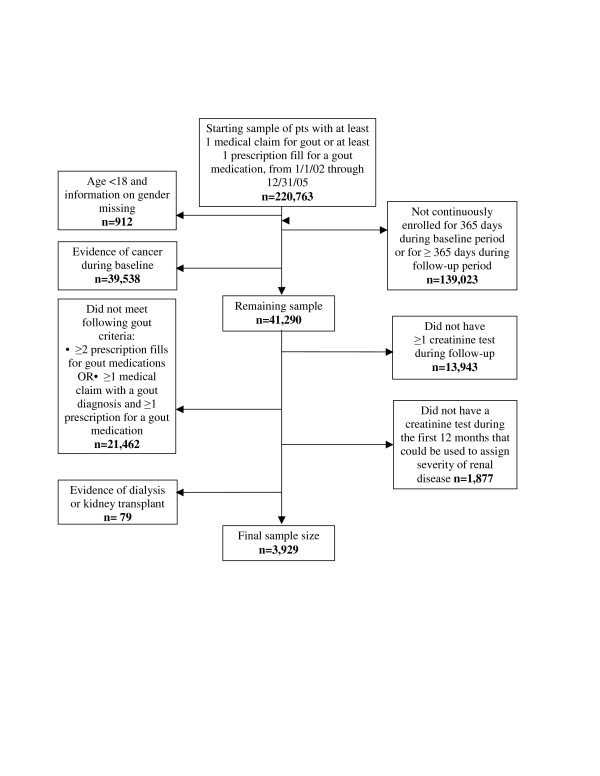
**Patient selection**. A total of 220,763 patients with at least 1 medical claim for gout or 1 fill for gout medication between 1/1/02 and 12/31/05 were initially identified from the health plan database. After application of all inclusion and exclusion criteria, a final sample of 3,929 patients was selected for the study.

A total of 220,763 patients were initially identified from the health plan database, based on the presence of at least 1 medical claim for gout or 1 fill for gout medication between 1/1/02 and 12/31/05 (Figure 1). After application of age criteria, continuous enrollment criteria, and criteria requiring no cancer during baseline, 41,290 patients remained. Of these 41,290 patients, 19,828 met the study criteria for having gout. The sample was further reduced to only include patients with available serum creatinine results during the first 12 months of follow-up. After application of all inclusion and exclusion criteria, a final sample of 3,929 patients was selected for the study.

Of the 3,929 patients who met the inclusion criteria, 1,536 (39%) were identified as having CKD. In a sensitivity analysis using the first serum creatinine measurement during the first 12 months (rather than the last measurement), subject distribution among CKD stages was similar. More than half (67.2%) of the subjects with CKD had Stage 2 CKD, 23.2% had Stage 3 CKD, and 9.6% had Stage 4 CKD (Table 1). CKD subjects were older (p < 0.0001) and more likely to be female (p = 0.0082) than those without CKD (Table 1). CKD patients had a greater number of comorbid conditions (p < 0.0001) and a higher Charlson-Deyo Comorbidity Index score (p < 0.0001) as compared to subjects without CKD. The five most common comorbid conditions observed in both patients with CKD and those without CKD were hypertension (67.6% of CKD patients vs. 48.5% of non-CKD patients); dyslipidemia (54.2% vs. 45.1%); non-gout arthropathies (35.4% vs. 34.4%); cardiovascular diseases (32.5% vs. 25.6%); and diabetes mellitus (25.8% vs. 16.4%). There was not a statistically significant difference in the length of follow-up between patients with CKD and those without CKD.

**Table 1:** Patient characteristics at baseline

Patient characteristics	No CKD (n)	No CKD (%)	Stage 2 CKD (n)	Stage 2 CKD (%)	Stage 3 CKD (n)	Stage 3 CKD (%)	Stage 4 CKD (n)	Stage 4 CKD (%)	p-value*
All patients	2,393	**-**	1,032	**-**	357	**-**	147	**-**	

Age group (years)									
≤44	858	35.85	138	13.37	38	10.64	33	22.45	< .0001
45-64	1,503	62.81	812	78.68	264	73.95	104	70.75	
65-74	31	1.3	79	7.66	48	13.45	9	6.12	
75+	1	0.04	3	0.29	7	1.96	1	0.68	

Male gender	2,121	88.63	896	86.82	300	84.03	120	81.63	0.0082

	**mean**	**SD**	**mean**	**SD**	**mean**	**SD**	**mean**	**SD**	

Number of comorbidities	6.85	4.81	7.51	5.33	9.68	5.83	8.19	5.53	< .0001

Charlson-Deyo Comorbidity Index score	0.44	0.87	0.55	0.94	1.25	1.43	1.33	1.66	< .0001

Age	47.83	9.02	53.71	8.5	57	8.74	51.52	9.81	< .0001

Length of follow-up (days)	912.68	449.7	927.12	455.37	896.94	428.41	936.73	467.52	0.634

eGFR (last value)	109.57	12.62	76.94	8.29	47.79	8.46	19.09	6.87	< .0001

SD = standard deviation.

### Serum uric acid levels and allopurinol use

A total of 3,122 gout patients used allopurinol at least once during the study period (79% of the study population). Only 77.5% of patients without CKD were prescribed allopurinol, compared to 80.0% of patients with Stage 2 CKD, 87.4% of patients with Stage 3 CKD, and 87.8% of patients with Stage 4 CKD. However, the mean follow-up serum uric acid level among allopurinol users with CKD was significantly higher than for allopurinol users without CKD (7.11 mg/dL vs. 6.75 mg/dL; p < 0.0001) (Table 2). Further, 25.6% of allopurinol users without CKD as compared to only 22.2% of allopurinol users with CKD reached the serum uric acid goal of < 6.0 mg/dL during the follow-up period (p = 0.0409).

**Table 2:** Serum uric acid lab results

Patient Characteristics		No CKD (N = 2,393)	Stage 2 CKD (N = 1,032)	Stage 3 CKD (N = 357)	Stage 4 CKD (N = 147)	p-value
Number of subjects with a lab result	**N**	1,542	645	220	73	

	**%**	64.44	62.5	61.62	49.66	0.0033

Value of last sUA test during follow-up period: all subjects	**mean**	6.86	7.09	7.56	7.72	< .0001
	**SD**	1.9	1.9	2.1	2.11	

Achieved goal of < 6 mg/dL based on last test during follow-up: all subjects	**n**	526	191	50	16	
	**%**	34.11	29.61	22.73	21.92	0.0008

Value of last sUA test during follow-up period: allopurinol users	**mean**	6.75	6.93	7.46	7.56	< .0001
	**SD**	1.91	1.85	2.11	2.05	

Achieved goal of < 6 mg/dL based on last test during follow-up: allopurinol users	**n**	448	172	49	16	
	**%**	25.64	23.31	20.16	18.82	0.1247

SD = standard deviation.

The mean initial dose of allopurinol decreased significantly with increasing severity of CKD (Table 3). Patients with Stage 2 CKD had a mean initial allopurinol dose of 249.0 mg/day, compared to 233.8 mg/day for patients with Stage 3 CKD and 217.6 mg/day for patients with Stage 4 CKD. The majority of patients who used allopurinol did not titrate their medication dose during the study period. Only about 14.6% of patients without CKD and 15.6% of patients with CKD had a dose titration. Although there was no significant difference in likelihood of dose titration between all patients with CKD vs. those without CKD (p = 0.45), patients with Stage 4 CKD were significantly more likely to have a dose titration compared to patients without CKD (p < 0.0001). Titration of dose upwards was more common than titration of dose downwards, both among patients with CKD and those without CKD (Table 3). We compared initial daily dose of allopurinol to the maintenance doses recommended by Hande et al. [9] using eGFR as a surrogate for creatinine clearance (Table 4). We found that 95.6% of subjects with an eGFR 100 or higher had an initial dose that did not exceed the recommended maintenance dose; however, this percentage was only 33.3% for eGFR 20-99, and 1.2% for eGFR0-19.

**Table 3:** Allopurinol use

Patient characteristics		No CKD (N = 2,393)	Stage 2 CKD (N = 1,032)	Stage 3 CKD (N = 357)	Stage 4 CKD (N = 147)	p-value
Used allopurinol during follow-up period, including index date	**N**	1,855	826	312	129	
	**%**	77.52	80.04	87.39	87.76	< .0001

Initial daily dose (mg)	**N**	1,855	826	312	129	
	**Mean**	248.17	249.02	233.78	217.63	0.0023
	**Std**	107.41	107.45	103.73	104.55	
	**Median**	300	300	300	200	

Last daily dose (mg)	**N**	1,552	699	260	109	
	**Mean**	268.93	261.63	248.08	241.54	0.0021
	**Std**	106.46	102.43	94.11	127.4	
	**Median**	300	300	300	300	

Difference between last dose and initial dose among subjects who titrated (mg)	**N**	269	108	53	36	
	**Mean**	106.02	102.73	66.26	86.33	0.5366
	**std**	192.07	188.55	167.93	187.99	

Subjects who titrated	**n**	270	108	53	36	
	**%**	14.56	13.08	16.99	27.91	0.00014

Increased between first and last dose	**n**	213	87	37	27	
	**%**	78.89	80.56	69.81	75	0.4253

Decreased between first and last dose	**n**	57	21	16	9	
	**%**	21.11	19.44	30.19	25	0.4253

**Table 4:** Adherence to allopurinol dosing guidelines

Hande et al. Creatinine Clearance	Hande et al. Criteria Maintenance Dose of Allopurinol	*Study Sample eGFR*	Study Sample Initial Daily Dose of Allopurinol	mg
0	100 mg every 3 days	0-9	n	22
			mean	240.91
			dose ≤ Hande maintenance dose (%)	0

10	100 mg every 2 days	10-19	n	60
			mean	233.14
			dose ≤ Hande maintenance dose (%)	1.67

20	100 mg daily	20-39	n	114
			mean	208.95
			dose ≤ Hande maintenance dose (%)	34.21

40	150 mg daily	40-59	n	245
			mean	236.35
			dose ≤ Hande maintenance dose (%)	29.8

60	200 mg daily	60-79	n	471
			mean	244.46
			dose ≤ Hande maintenance dose (%)	35.46

80	250 mg daily	80-99	n	892
			mean	249.58
			dose ≤ Hande maintenance dose (%)	32.96

100	300 mg daily	100-119	n	909
			mean	247.34
			dose ≤ Hande maintenance dose (%)	95.93

120	350 mg daily	120-139	n	389
			mean	253.09
			dose ≤ Hande maintenance dose (%)	94.86

140	400 mg daily	140+	n	20
			mean	250
			dose ≤ Hande maintenance dose (%)	95

Multivariable logistic regression analysis was used to assess factors associated with titration (Table 5). Adjusting for covariates, patients with severe CKD had a significantly higher likelihood of dose titration compared to allopurinol users without CKD (odds ratio [OR] 2.130, 95% confidence interval [95% CI]: 1.380 - 3.289). Other factors associated with dose titration were thiazide use during follow-up (OR 1.301, 95% CI 1.035 - 1.635) and a higher pre-index Charlson-Deyo Comorbidity Index score (OR 1.164, 95% CI 1.059 - 1.279). Increased age was associated with a decreased odds of dose titration (OR 0.973, 95% CI 0.962 - 0.985). Results using a threshold for titration of ≥100 mg were similar.

**Table 5:** Multivariate models predicting an average allopurinol dose change ≥50 mg/day

	Odds Ratio	95% Confidence Interval Lower limit	95% Confidence Interval Upper limit	p-value
Degree of renal impairment				
Stage 2 CKD*	0.939	0.729	-1.208	0.623
Stage 3 CKD*	1.218	0.857	-1.732	0.2723
Stage 4 CKD*	2.13	1.38	-3.289	0.0006

Age	0.973	0.962	-0.985	< .0001

Male gender	0.948	0.686	-1.309	0.7455

Geographic region**				
West	1.303	0.83	-2.045	0.2507
South	1.314	1.019	-1.693	0.0353
Northeast	1.641	1.141	-2.362	0.0076

Thiazide use during follow-up	1.301	1.035	-1.635	0.0244

Charlson-Deyo Comorbidity Index score	1.164	1.059	-1.279	0.0016

Months of allopurinol use	1.022	1.015	-1.028	< .0001

*The reference group is patients with no CKD.

** The reference group is the Midwest

## Discussion

The prevalence of CKD in the US general population is approximately 13% [3]. This retrospective study reveals a very high prevalence of CKD (approximately 40%) among gout patients in the US. A previous smaller study of gout patients in New Zealand found a level of CKD prevalence similar to what is reported here [25]. In the present study, gout patients with severe renal disease were somewhat older and more likely to be female and to have a greater overall comorbidity burden. As expected, serum uric acid concentrations were higher among those with worse renal disease. Although individuals with CKD were treated more frequently, they received lower initial doses of allopurinol. Over time, the average allopurinol dose among patients with gout increased regardless of the presence or severity of renal disease. The magnitude of increase among those with renal disease was smaller but statistically indistinguishable from those with normal renal function. Thus, the treatment received by those with normal renal function was not substantially more aggressive than the treatment received by those with impaired renal function.

Within each cohort, patients who used allopurinol had lower serum uric acid levels than patients who did not use allopurinol. However, a significant finding of this study was the relatively low proportion of patients treated with allopurinol who achieved a serum uric acid concentration of < 6 mg/dL (25.6% of patients without CKD, 23.3% of those with Stage 2 CKD, 20.2% of those with Stage 3 CKD, and 18.8% of those with Stage 4 CKD). Although goal attainment was low among all patient cohorts, presence of CKD decreased the likelihood of meeting this benchmark even further. Further, a low percentage of patients with CKD (only about 16%) had their allopurinol dosage titrated. These findings highlight the therapeutic challenge of balancing the risks and benefits of administering allopurinol to patients with impaired renal function.

Appropriate dosing of allopurinol when CKD is present is an important yet controversial topic. The US Food and Drug Administration has approved the following dosing regimen for gout without concurrent renal disease [26]: Start with a dose of 100 mg/day and increase by 100 mg/day at weekly intervals until the desired therapeutic response is achieved. A maximum dose of 800 mg/day is permitted. However, in a Japanese study, patients achieved a therapeutic serum concentration of oxypurinol (> 4.6 mcg/mL) and a clinical response rate of > 90% at an allopurinol dose of just 100-200 mg/day [27]. Dosing in this study was based on levels of serum creatinine, not on eGFR. Others have raised concerns about the safety of higher doses of allopurinol, and using serum creatinine as an index for allopurinol dosing has been shown to be less safe than using eGFR [28]. Adhering to the current guidelines for dosing allopurinol based on eGFR, on the other hand, does not necessarily result in desired uric acid control [9,29,30]. The results of the managed care analysis performed here reflect this therapeutic challenge.

Our findings do not indicate that initial daily dose of allopurinol is prescribed in a manner that correlates with published guidelines sensitive to renal function. The original guidelines for allopurinol dosing were based on an extensive review of severe adverse reactions to allopurinol and a small *de novo *study of 40 patients [9]. That study calculated that renal clearance of oxypurinol was directly proportional to the creatinine clearance, which was calculated using the Cockroft-Gault formula. The recommended dosing levels were based on achieving a target serum oxypurinol level of 30-100 mmol/L and not on avoiding any toxicity threshold. In another study based on data from a rheumatology practice, there were modest correlations between allopurinol doses and plasma oxypurinol concentrations, and between creatinine clearance and plasma oxypurinol [30]. However, there was no correlation between plasma oxypurinol and plasma uric acid, and only 50% of patients within the therapeutic range of plasma oxypurinol had a plasma uric acid level < 0.42 mmol/L [30]. Collectively, these findings suggest that there is considerable heterogeneity in drug handling, and future studies need to test the underlying assumption that oxypurinol is the sole active metabolite responsible for therapeutic efficacy and toxicity. Concerns regarding adverse events in particular may influence the aggressiveness of current treatment regimens. Cutaneous adverse reactions to allopurinol occur in approximately 7.7 per 1000 recipients [31], and the incidence of allopurinol hypersensitivity syndrome is about 2-3 times higher among renally impaired patients compared to non-renally impaired patients [32]. However, the relationship between adverse events and allopurinol dosing is still not fully understood. Previous studies have shown that patients receiving doses of allopurinol above the recommended dose (as based on the creatinine clearance rate) did not exhibit increased toxicity [33,34]. Other studies have indicated that specific genetic markers may influence the risk of experiencing severe cutaneous adverse reactions following allopurinol treatment [35].

A few caveats must be considered when interpreting the results of the present study: (1) The approach used to identify gout patients from claims data is subject to misclassification errors. This problem was addressed by performing sensitivity analyses that specified different combinations of coding, medications, and laboratory criteria as alternate selection criteria. The resulting patient populations varied in terms of size and other characteristics, but the fundamental findings in this analysis held. (2) Because of the requirement of availability of a serum creatinine test, the current study population may have been less renally healthy than other populations of gout patients. This factor could reduce the generalizability of the findings. (3) The authors studied patients with gout seeking medical care, thereby potentially selecting those with more severe or frequent flare-ups of gout; this, in turn, may have overestimated the prevalence of CKD among gout patients. (4) The multivariate model examining allopurinol dose change did not distinguish between dose increases or decreases. This would be a valuable direction for future research. (5) A limitation of claims analysis is that we are only able to measure prescribed doses, which may differ from the dose that a patient actually took.

## Conclusion

In this study a high prevalence of CKD was observed among gout patients. Serum uric acid goal attainment was low among patients treated with allopurinol, and poorest among those with CKD. The findings suggest that poor outcomes among gout patients with CKD are partly due to clinicians' reluctance to prescribe higher doses of allopurinol to patients with impaired renal function. The benefits and risks of allopurinol treatment must be weighed carefully in patients with CKD, and alternate treatment approaches are needed to improve the prognosis of these patients. Future research should address at least three additional issues: (1) the role of non-oxypurinol metabolites of allopurinol in efficacy and toxicity, (2) more sophisticated pharmacogenomics-based studies of allopurinol dosing in the presence of CKD, and (3) development of urate-lowering drugs that are not renally cleared. Additionally, our results suggest a need to raise awareness among physicians regarding the importance of titrating therapy to reach uric acid goals.

## List of Abbreviations

AHRQ: Agency for Healthcare Research and Quality; CKD: chronic kidney disease; eGFR: glomerular filtration rate; HIPAA: Health Insurance Portability and Accountability Act; ICD-9-CM: International Classification of Diseases, Ninth Revision, Clinical Modification; MDRD: Modification of Diet in Renal Disease; NKF KDOQI: National Kidney Foundation Kidney Disease Outcomes Quality Initiative; sUA: serum uric acid

## Competing interests

This study was funded by Takeda Pharmaceuticals International, Inc. (TPI), Deerfield, IL. (TAP Pharmaceutical Products Inc. is now a part of TPI.) Dr. Krishnan has received grant support from Takeda Pharmaceuticals International, Inc. (TPI), and has held stock in Savient Pharmaceuticals. He has served as an advisor/consultant for both of these companies. Proprietary products manufactured by these companies are not named/discussed in this manuscript. Dr Krishnan is supported in part from a Research Starter Grant from the PhRMA Foundation and a career development grant from the American College of Rheumatology Research and Education Foundation. Dr. Pandya is an employee of Takeda Pharmaceuticals International, Inc., and Dr Fuldeore was previously employed by TAP Pharmaceutical Products, Inc., and now works for Abbott Laboratories. Drs Riedel and Zarotsky are employees of OptumInsight.

## Authors' contributions

MF, AR, VZ, BP, OD, and EK participated in the design of the study and helped to draft the manuscript. All authors have read and approved this manuscript.

## Pre-publication history

The pre-publication history for this paper can be accessed here:

http://www.biomedcentral.com/1471-2369/12/36/prepub
